# *TP53*: the unluckiest of genes?

**DOI:** 10.1038/s41418-024-01391-6

**Published:** 2024-10-23

**Authors:** Andreas C. Joerger, Thorsten Stiewe, Thierry Soussi

**Affiliations:** 1 Institute of Pharmaceutical Chemistry, Goethe University, Frankfurt am Main, Germany; 2 Structural Genomics Consortium (SGC), Buchmann Institute for Molecular Life Sciences, Frankfurt am Main, Germany; 3 Institute of Molecular Oncology, Universities of Giessen and Marburg Lung Center (UGMLC), German Center for Lung Research (DZL), Philipps University, Marburg, Germany; 4 Institute for Lung Health (ILH), Justus Liebig University, Giessen, Germany; 5 Equipe « Hematopoietic and Leukemic Development », Sorbonne Université, INSERM, Centre de Recherche Saint-Antoine, CRSA, AP-HP, SIRIC CURAMUS, Paris, France; 6 Dept. of Immunology, Genetics and Pathology, Science for Life Laboratory, Uppsala University, Clinical Genetics, Uppsala University Hospital, Uppsala, Sweden

**Keywords:** Gene regulation, Protein folding, Tumour-suppressor proteins

## Abstract

The transcription factor p53 plays a key role in the cellular defense against cancer development. It is inactivated in virtually every tumor, and in every second tumor this inactivation is due to a mutation in the *TP53* gene. In this perspective, we show that this diverse mutational spectrum is unique among all other cancer-associated proteins and discuss what drives the selection of *TP53* mutations in cancer. We highlight that several factors conspire to make the p53 protein particularly vulnerable to inactivation by the mutations that constantly plague our genome. It appears that the *TP53* gene has emerged as a victim of its own evolutionary past that shaped its structure and function towards a pluripotent tumor suppressor, but came with an increased structural fragility of its DNA-binding domain. *TP53* loss of function - with associated dominant-negative effects - is the main mechanism that will impair *TP53* tumor suppressive function, regardless of whether a neomorphic phenotype is associated with some of these variants.

## Introduction—Mutational patterns in cancer-associated proteins

Established more than 30 years ago, the concept that tumor development relies on the activation of oncogenes and the loss of function of tumor suppressor genes is still prevailing, but this binary view needs revising as genes associated with DNA replication and repair or immune escape were shown to be important players as well [1–5].

Alterations in oncogenes associated with hyperactivity (hypermorphic variants) or with a gain of function (neomorphic variants) are usually single nucleotide substitutions (SNS) creating missense variants in the functional domain of the protein. These oncogenic mutations, therefore, have a skewed distribution along the protein sequence and cluster at very specific positions. The limited number of hotspot variants in oncogenes such as KRAS (codons 12 and 13), the phosphatidylinositol 3-kinase catalytic subunit, PIK3CA (codons 542 or 545), BRAF (codon 600), EGFR (codon 858), isocitrate dehydrogenase 1, IDH1 (codon 132) or Kit (codon 816) are perfect examples of this restriction [6]. This observation is also supported by multiple saturation mutagenesis analysis showing that cancer-associated variants in oncogenes are restricted to a few residues [7].

In contrast, loss-of-function alterations in tumor suppressor genes (amorphous or hypomorphic variants) are predominantly events that will prevent protein expression such as indels, splicing or nonsense mutations that can be distributed across the protein sequence. This is reflected by the great diversity of some of those events (indels of different sizes) and their large distribution along the protein for most tumor suppressor genes. Fortunately, indels occur far less frequently than missense mutations in both normal or tumoral genomes by about one order of magnitude (Supplementary Fig. 1) [8, 9]. Furthermore, as both alleles of these genes need to be inactivated, this lowers the probability for the selection of a pathogenic loss of function.

**Fig. 1:**
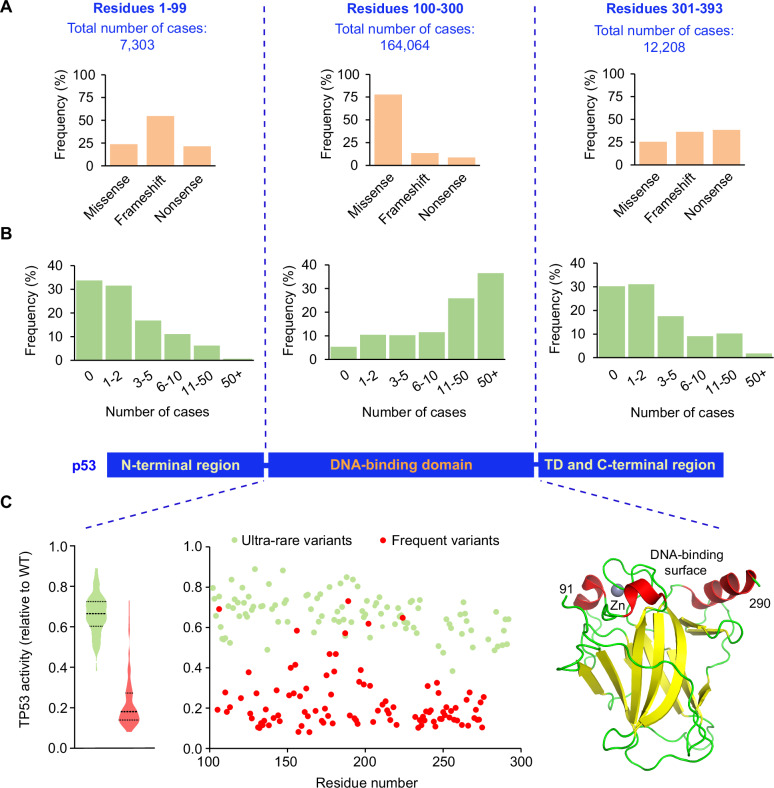
Analysis of somatic *TP53* variants in tumor tissues according to their frequency in the UMD_TP53 database (2024 release, 248,363 patients). **A** Somatic missense variants are localized predominantly in the DNA-binding domain (central panel) of the multidomain p53 protein and have a much lower frequency in the flanking N- and C-terminal regions (left and right panel, respectively). **B** Frequent cancer-associated somatic variants are found predominantly in the DNA-binding domain, whereas low frequency variants are more predominant in the N- and C-terminal regions and correspond to indels. TD = tetramerization domain (**C**) *TP53* loss of activity for missense variants localized in the DNA-binding domain of the p53 protein. p53 activities ranging from 0 (inactive) to 1 (full activity) were taken from the work of Kotler et al. [39]. Red circles: variants frequently found in human cancer (at least 75 cases in the database); green circles: ultra rare variants (1 or 2 cases) or variants that have not been reported in cancer yet. For each residue, the average activity of all frequent or rare variants is given. Violin plots illustrate the distribution of *TP53* activity for the two classes of *TP53* variants. The structure of the DNA-binding domain is shown on the right as a cartoon representation (PDB entry 2XWR) [77].

A single gene, the so-called *TP53* tumor suppressor gene, does not fit into this picture as the vast majority of mutational events are missense mutations spread across more than half of the protein. Missense mutations predominate in the central DNA-binding domain (DBD), whereas frameshift variants are more frequent in the N- and C-terminal regions of the protein (Fig. 1A, B) [10–12]. It is generally assumed that this very specific mutational landscape in *TP53* is linked to a specific selection of variants with a potential gain of novel antimorphic or neomorphic functions [10, 13]. Without excluding the possibility that the high expression of heterogenous missense p53 variants has a clinical relevance in human tumors, we show below that this mutational landscape is predominantly shaped by the extreme fragility of the p53 protein, as demonstrated by systematic functional analyses and structural studies.

## Early discoveries hinting at p53’s structural fragility and its implications

The first hint that p53 mutants sustain a change of conformation originated from the observation that they could be recognized by the Hsc70/Hsp70 chaperone [14, 15]. In addition, some p53 monoclonal antibodies were shown to react with alternative conformational forms of p53 [16]. Generation and epitope mapping of novel monoclonal antibodies revealed that this conformational flexibility affects the central region of the protein, which was not yet defined as a DBD at the time [17, 18]. Functional analysis of the major *TP53* hotspot variants revealed that *TP53* variants with mutations at codon 175, such as R175H or R175P, were associated with an unfolded conformation and chaperone binding, whereas variations at codon 248 or 273 (e.g., R248L or R273H) did not induce any change [19]. The elucidation of the crystal structure of the central region of p53 and the identification of this domain as a specific DBD then provided an explanation for the above findings and led to this simple binary classification of common cancer mutations as contact mutations that disrupt amino acids directly contacting DNA (at codons 273 and 248) and structural mutations that destabilize the structure/conformation of the DBD (at codons 175, 249, or 282) [20–22]. As it later turned out, the R175H variant actually belongs to a special subgroup of structural mutations that directly impair zinc binding, which is key for the overall thermodynamic and kinetic stability of the DBD and the structural integrity of the DNA-binding surface [23–25]. Further evidence for the intrinsic instability of p53 came from the discovery of thermosensitive variants in both mouse and human cells, a very rare observation for a mammalian gene [26, 27]. These variants, such as the A138V and V143A mutants initially studied (human numbering), have a wild-type-like conformation and activity at permissive temperature (32 °C), whereas the protein unfolds when shifted to non-permissive temperature (37 °C), leading to a loss of function and an association with the chaperone Hsc70. Since then, many more temperature-sensitive p53 cancer mutants have been identified and characterized [28–31]. A similar behavior upon temperature shift was also found for the p53 homolog from the cold-blooded clawed frog *Xenopus laevis*, although in that case for the wild-type protein. When expressed at 37 °C, a temperature above the physiological body temperature of the animal, the *X. laevis* p53 DBD adopts a mutant-like conformation, binds to chaperone Hsc70, and the full-length protein loses all its growth suppressive activity, whereas it behaves normally at 32 °C or below [32, 33]. A similar observation has been reported for Drosophila p53 [34].

The above observations on the p53 folding state in cells are consistent with structural and biophysical studies on the p53 protein that described its DBD as intrinsically unstable [35]. This domain is therefore very sensitive to local perturbation induced by any mutation, leading in many cases to global unfolding at body temperature, which is accompanied by the loss of its DNA-binding ability [23]. In other words, its low intrinsic stability makes p53 susceptible to inactivation by destabilizing mutations that would result in a benign phenotype in the context of a more stable structural scaffold [36].

## Insights from large-scale functional analyses of *TP53* variants

**Fig. 2:**
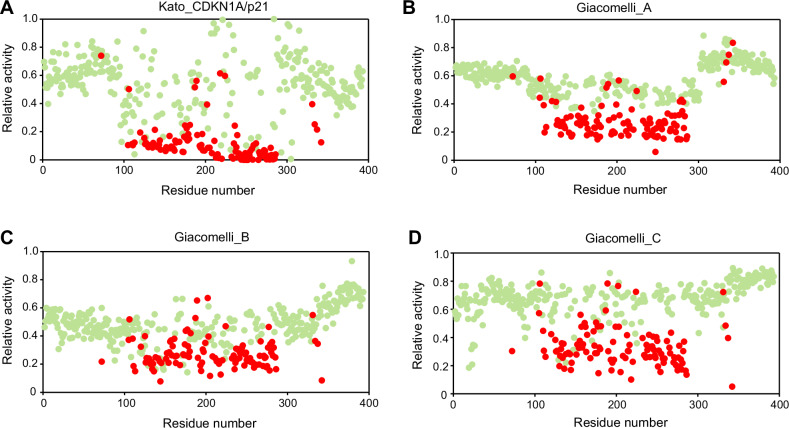
Distribution of loss-of-activity missense variants in the p53 protein. p53 activities ranging from 0 (inactive) to 1 (full activity) were taken from the work of Kato et al. (CDKN1A/p21 promoter) [37] (**A**) or from the three different readouts described by Giacomelli et al. [40] (**B**–**D**). Mutation frequencies in tumors (somatic count) are taken from the upcoming release of the UMD_TP53 database (2024 release, 248,363 patients). Red circles: variants frequently found in human cancer (more than 75 cases in the database); green circles: ultra rare variants (only 1 or 2 cases in the database) or variants that have never been reported in cancer. For each residue, the average activity of all frequent or rare variants is given. The outlier at codon 72 is due to the R72P polymorphism.

In a landmark paper published by the group of C. Ishioka in 2003, the authors performed a saturation mutagenesis of every *TP53* position and demonstrated that most missense mutations in the central DBD of *TP53* lead to a loss of the transactivation activity of the protein [37]. There is a strong correlation between the mutations found in human tumors and inactive variants from this artificial library [38]. Recent studies have extended these observations with the analysis of *TP53* saturation mutagenesis screens in mammalian cells, using either cell death or growth arrest as a readout of *TP53* activity [39–41]. One of the great strengths of saturation mutagenesis of *TP53* is the unbiased generation of variants, irrespective of whether they were selected in human cancer. These three studies highlighted two important features: First, in stark contrast to other tumor suppressor genes, more than half of all possible *TP53* missense variants in the DBD lead to a loss of function that for most missense and indel variants is indistinguishable. This alleviates the need to select for less frequent indel mutations, which is supported by the observation that the frequency of frameshift *TP53* mutations in human cancer (10–15%) is similar to the frequency observed in the human genome (Supplementary Fig. S1). Second, whatever assays have been used to record *TP53* loss of function, missense variants that are not found in human cancer usually displayed a significant residual activity (Figs. 1C and 2). Outliers, i.e., missense mutations that are recurrently found in cancer but failed to show evidence for loss of function, often induce splice alterations as revealed by RNA analysis of tumor tissues or knock-in screens [41, 42]. Together, the strong correlation of loss of function and occurrence in cancer emphasizes the need for a loss of function to generate variants selected during neoplasia transformation.

## Several dominant-negative effect mechanisms exacerbate the effects of loss-of-function mutations

Compared with other tumor suppressors, such as Retinoblastoma protein or APC, p53 is at a further disadvantage when it comes to inactivation by mutation: it forms tetramers [43, 44]. As shown initially for the test case of the DNA-contact mutant R273H and later by saturating mutagenesis, missense mutants of the p53 DBD, but not outside of it, exert a dominant-negative effect over the wild-type protein through the formation of mixed tetramers with weakened DNA-binding [45, 46], which drastically reduces the amount of fully active wild-type homotetramers in heterozygous cells to a level that may not be sufficient for efficient tumor suppression.

The cell’s misery is compounded by the fact that p53 exposes a series of aggregation-prone sequence motifs upon unfolding. As if opening Pandora’s box, unfolding of thermolabile mutants unleashes a wave of destruction, resulting in coaggregation not only with the wild-type protein in heterozygous cells but also with other cellular proteins, including the paralogs p63 and p73 [47]. DBD-mediated coaggregation with the tumor suppressor p73 effectively kills off a potential salvage pathway [33, 48–50], and binding to p73 was postulated to be one of the possible gain-of-function mechanisms of mutant p53 [51, 52], although this is in essence a dominant-negative effect. So at least some neomorphic phenotypes may be directly linked to p53-mediated aggregation, in addition to the mere loss of p53 transcriptional activity. From a molecular viewpoint, many so-called gain of function phenotypes may be alternatively viewed as the phenotypic manifestation of a loss of multiple functions rather than the creation of a mutant protein with novel functions.

Another important aspect is that there is an overabundance of p53 protein in p53-mutated tumor cells, due to perturbation of its normal proteasomal degradation pathways (usually referred to as stabilization of mutant p53 - but not to be confused with the conformational stability of the protein discussed elsewhere in this perspective) [53]. Dysregulated, highly abundant mutant p53 proteins have the potential to exert an active role in tumor cells, not only via DBD-mediated coaggregation but also via their promiscuous interaction modules in the disordered N- and C-terminal regions, and this may be one of the contributing factors why mutant p53 tumors have a poorer prognosis than wild-type tumors in certain cancers [54, 55]. However, the overabundance of mutant p53 protein in tumors is usually directly linked to secondary genetic alterations such as aneuploidy and loss of p16INK4A or Mdm2 [56–59], in addition to *TP53* loss of function, and may therefore contribute to tumor progression only at later stages [41]. Moreover, p53 transcriptional activity serves as a more effective stratifier of cancer patient outcomes than mutation status [10–12], suggesting that prognosis is primarily driven by p53 loss of function.

## What drives the selection of *TP53* mutations in cancer?

Based on the above observations, all deleterious *TP53* missense variants in the DBD would be expected with a similar prevalence in cancer patients. However, the *TP53* mutation spectrum shows clear mutational hotspots, somewhat reminiscent of the mutation profile of classical oncogenes. While this might suggest that some variants display a specific oncogenic gain of function that is actively selected in cancers, there are alternative explanations. First, the mutational hotspots are characterized by DNA sequences with elevated intrinsic susceptibility to the most prevailing mutagenic mechanisms that drive genomic instability in cancer cells [40]. For example, the six most frequent missense variants occur at methylated CpG sites that are particularly prone to aging-related mutagenic processes, and the R249S hotspot mutation, which is prevalent in hepatocellular carcinoma in sub-Saharan Africa and Southeast Asia, has been directly linked to exposure to the crop contaminant aflatoxin B1 [60]. Importantly, the combination of functional data obtained by saturating mutagenesis with mutational probabilities derived from the most common mutagenic processes in cancer provided a near-perfect model of the observed spectrum of *TP53* missense variants, without the need to postulate additional oncogenic gain-of-function effects [40]. Second, unlike most indel mutations, missense mutations produce mutant peptides that can be recognized by the immune system as neoantigens [61]. These neoantigens have the therapeutic potential to be utilized in directing immune responses against the tumor [62]. Interestingly, the most prevalent hotspot mutants seem to be less immunogenic than non-hotspot mutants [63]. Insufficient clearance of poorly antigenic variants by the immune system may therefore further contribute to their prevalence in cancer samples.

Therefore, in contrast to oncogenes that have a limited number of residues that can lead to a gain of function and tumor suppressor genes that need more drastic modifications such as indel events to display a loss of function, 55% of potential missense variants that can occur in the DBD of the p53 protein have been found in human cancer (at least 10x) and display a loss of activity, either complete or partial. Prevalence differences between individual missense variants are explained well by mutational probabilities and immunogenic properties. *TP53* loss of function (and associated dominant-negative effects) remains the main mechanism that will impair *TP53* tumor suppressive function, regardless of whether a neomorphic phenotype is associated with some of these variants. In support of this, a recent study by Wang et al. demonstrated that removal of mutant p53 genes that had been associated with oncogenic gain-of-function properties by CRISPR/Cas9 had no effect on cancer cell growth in vitro or in vivo [64, 65].

It is important to consider that the frequency of *TP53* mutations differs widely between different tumor types [11, 12, 66] and that the inherent genetic background of the individual could also play a role [67, 68]. For instance, *TP53* mutations are almost universal in small-cell lung cancer, which originates from rare neuroendocrine lung cells, but are much less frequent in lung adenocarcinomas, which arise from alveolar type II or club cells. Additionally, mutant p53 can enhance the activity of co-mutated proto-oncogenes. For example, by inducing the splicing regulator hnRNPK, mutant p53 alters the splicing of mRNAs encoding GTPase-activating proteins, resulting in heightened KRAS signaling [69]. Conversely, oncogenic KRAS signaling increases CREB1 phosphorylation, enabling its binding to mutant p53. Together, they hyperactivate the expression of the pro-metastatic transcription factor FOXA1 [70]. Thus, the selection of *TP53* mutations in cancer is strongly influenced by cell- or tissue-specific factors and the presence of cooperating oncogenes.

## *TP53*: a victim of its evolutionary past?

It appears that the diversity of oncogenic *TP53* variants is predominantly due to the misfortune of *TP53* to encode an excessively fragile and extended functional domain that can be easily targeted by the high frequency of SNS that plague normal and tumor cells. Although in vitro analyses and mouse models suggest various heterogenous antimorphic or neomorphic activities for several variants, confirmation of the clinical relevance of these findings is still pending.

Protein structures are usually remarkably robust to changes in the primary sequence, but they have evolved for optimal function in cells and not necessarily the greatest stability [71, 72]. The extreme fragility of p53 may therefore be necessary to provide the structural plasticity to adapt its conformation to sense and react to local perturbation in the cellular environment, and also allow for rapid p53 turnover [73, 74]. Intriguingly, unlike its more stable paralogs, p63 and p73, which have retained more ancestral features, the p53 protein appears to have evolved at a much faster rate, which also affected its stability [74]. In vertebrates, there is a good correlation between the thermodynamic stability of the p53 DBD and the body temperature of a particular organism or the optimal temperature of the habitat in the case of cold-blooded animals, suggesting that human p53 may have evolved to be only marginally stable and now pays the price for its evolutionary past when faced with mutations [73–76]. Unlucky, indeed.

## Supplementary information

Supporting Information
