# Supplementary material for: TP53: the unluckiest of genes?
Source: Cell Death Differ. 2024 Oct 23;32(2):219–24. doi: 10.1038/s41418-024-01391-6 (PMC11803090; doi:10.1038/s41418-024-01391-6)
Supplement: Supplementary file 1 — Supporting Information [file 41418_2024_1391_MOESM1_ESM.pdf]

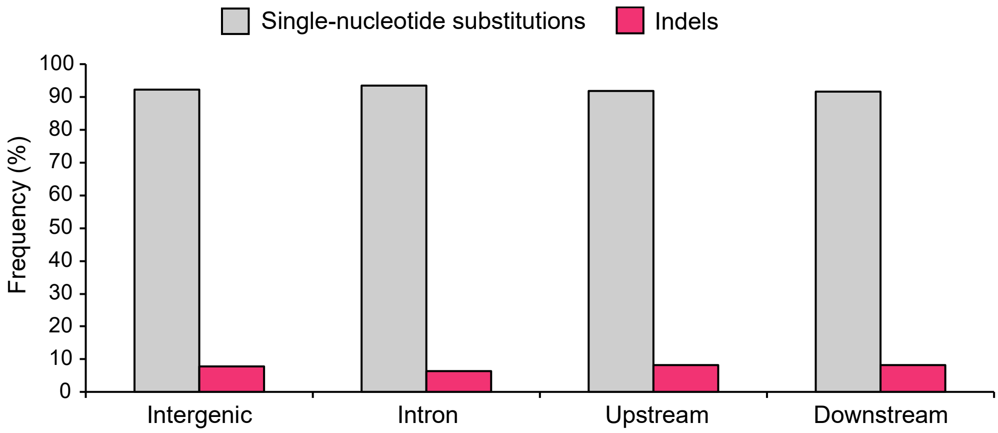

Supplementary Figure S1. Relative frequency of single-nucleotide substitutions (SNS) and indels in various non-coding regions of the genome of 2809 tumors analyzed by whole genome sequencing (PCAWG study available at https://xenabrowser.net/datapages/)
